# Memory SARS-CoV-2 T-cell response in convalescent COVID-19 patients with undetectable specific IgG antibodies: a comparative study

**DOI:** 10.3389/fimmu.2023.1142918

**Published:** 2023-04-26

**Authors:** Raquel Fernández-Moreno, Jorge Valle-Arroyo, Aurora Páez-Vega, Ana Salinas, Angela Cano, Ana B. Pérez, Julián Torre-Cisneros, Sara Cantisán

**Affiliations:** ^1^ Spanish Network for Research in Infectious Diseases (REIPI), Centro de Investigación Biomédica en Red de Enfermedades Infecciosas (CIBERINFEC), Instituto de Salud Carlos III, Madrid, Spain; ^2^ Infectious Diseases (GC-03) and Clinical and Molecular Microbiology (GC-24) Groups, Maimonides Biomedical Research Institute of Cordoba (IMIBIC), Reina Sofía University Hospital, University of Cordoba, Cordoba, Spain; ^3^ Infectious Diseases Unit, Reina Sofía University Hospital, Cordoba, Spain; ^4^ Microbiology Unit, Reina Sofía University Hospital, Cordoba, Spain

**Keywords:** COVID-19, FASCIA assay, SARS-CoV-2, undetectable SARS-CoV-2 IgG, proliferative T-cell response

## Abstract

**Background:**

During the COVID-19 pandemic, a variable percentage of patients with SARS-CoV-2 infection failed to elicit humoral response. This study investigates whether patients with undetectable SARS-CoV-2 IgG are able to generate SARS-CoV-2 memory T cells with proliferative capacity upon stimulation.

**Methods:**

This cross-sectional study was conducted with convalescent COVID-19 patients, diagnosed with a positive real-time PCR (RT-PCR) from nasal and pharyngeal swab specimens. COVID-19 patients were enrolled ≥3 months after the last PCR positive. Proliferative T-cell response after whole blood stimulation was assessed using the FASCIA assay.

**Results:**

A total of 119 participants (86 PCR-confirmed COVID-19 patients and 33 healthy controls) were randomly filtered from an initial cohort. Of these 86 patients, 59 had detectable (seropositive) and 27 had undetectable (seronegative) SARS-CoV-2 IgG. Seropositive patients were subclassified as asymptomatic/mild or severe according to the oxygen supplementation requirement. SARS-CoV-2 CD3+ and CD4+ T cells showed significantly lower proliferative response in seronegative than in seropositive patients. The ROC curve analysis indicated that ≥ 5 CD4+ blasts/μL of blood defined a “positive SARS-CoV-2 T cell response”. According to this cut-off, 93.2% of seropositive patients had a positive T-cell response compared to 50% of seronegative patients and 20% of negative controls (chi-square; p < 0.001).

**Conclusions:**

This proliferative assay is useful not only to discriminate convalescent patients from negative controls, but also to distinguish seropositive patients from those with undetectable SARS-CoV-2 IgG antibodies. Memory T cells in seronegative patients are able to respond to SARSCoV-2 peptides, although at a lower magnitude than seropositive patients.

## 1 Introduction

Coronavirus disease 2019 (COVID-19) is an infectious disease caused by severe acute respiratory syndrome coronavirus 2 (SARS-CoV-2). To date, more than 500 million cases of confirmed SARS-CoV-2 infection and 6.4 million deaths from COVID-19 have been reported to the World Health Organization (1). Most COVID-19 patients remain asymptomatic or experience mild symptoms, whereas approximately 15–20% progress to more severe disease, which can lead to acute respiratory distress syndrome, respiratory failure and eventually death (2). Since the outbreak of the COVID-19 pandemic, immune response against SARS-CoV-2 has been widely studied and many publications have attempted to identify the immune profile associated with disease severity (3). Depending on the severity of coronavirus disease, significant differences in the level of antibodies or T-cell response have been reported in the literature (4–9).

During the natural course of SARS-CoV-2 infection a small percentage of patients failed to elicit humoral response (10, 11). However, the reason these individuals lacked specific IgG response is unclear. Did they have a low viral load that was insufficient to trigger the development of SARS-CoV-2 IgG? Do these patients belong to a transient group that will finally convert to positive SARS-CoV-2 IgG? And more importantly, is SARS-CoV-2 T-cell response induced in these seronegative patients?

To address these questions, we compared SARS-CoV-2 T-cell response between seropositive and seronegative convalescent COVID-19 patients. For this purpose, we used the Flow-cytometric Assay for Specific Cell-mediated Immune-response in Activated whole blood (FASCIA). This assay is a well-established method for assessing T-cell proliferative reactivity against different stimuli in an easy-to-use and cost-effective format based on whole blood (12).

## 2 Material and methods

### 2.1 Study population and design

This cross-sectional study was conducted with convalescent COVID-19 patients at the Reina Sofia University Hospital of Cordoba, Spain. Adult COVID-19 convalescent patients who met the following inclusion criteria were eligible for the study: 1) patients diagnosed with a positive real-time PCR (RT-PCR) from nasal and pharyngeal swab specimens in May–December 2020; 2) patients in which IgG anti-SARS-CoV-2 was determined after the PCR. The patients were enrolled between February and June 2021. Non-COVID-19 healthy controls (RT-PCR and anti-SARS-CoV-2 IgG negative) were also enrolled in the study. Peripheral blood samples were collected from all participants and anti-SARS-CoV-2 IgG was re-tested in plasma samples. In the case of patients, blood samples were taken ≥ 3 months after the last positive SARS-CoV-2 RT-PCR. None of the study participants had received the COVID-19 vaccine at the time of recruitment. Informed consent approved by the Institutional Review Board was obtained from all participants. The study was conducted in accordance with the Declaration of Helsinki and the Ethics Committee (Institutional Review Board) of the Reina Sofia University Hospital (Code 4800) approved the protocol.

### 2.2 Grouping criteria

COVID-19 patients and negative controls were randomly filtered from an initial cohort and classified into four age- and gender-matched groups according to the SARS-CoV-2 RT-PCR and anti-SARS-CoV-2 IgG test results as well as the oxygen supplementation requirement, as follows: 1) seropositive patients (PCRpos IgGpos) with asymptomatic/mild infection, not requiring oxygen supplementation; 2) seropositive patients (PCRpos IgGpos) with severe disease who received oxygen supplementation; 3) seronegative patients (PCRpos IgGneg) and 4) healthy controls (PCRneg IgGneg).

### 2.3 Serological assays

For the initial classification of the groups of patients, anti-SARS-CoV-2 IgG determination was performed in clinical routine using an indirect chemiluminescent immunoassay (CLIA) (COVID-19 Virclia^®^ IgG monotest, Vircell Microbiologists, Spain). At the time of blood sample collection, anti-SARS-CoV-2 IgG was re-tested on plasma using a quantitative chemiluminescence immunoassay (CLIA) (Liaison SARS-CoV-2 TrimericS IgG assay, Diasorin) according to the manufacturer’s instructions. IgG antibody levels were expressed as binding antibody units per mL (BAU/mL). A result of ≥ 33.8 BAU/mL was considered positive.

Additionally, SARS-CoV-2 IgM and IgA antibody levels were analyzed at this time. IgM determination was performed using a qualitative chemiluminescence immunoassay (CLIA) (Liason SARS-CoV-2 IgM, Diasorin) and they were expressed as an index value. An index result of ≥ 1.10 (positive) generally indicates the presence of IgM antibodies against SARS-CoV-2 and exposure to the virus. IgA antibody levels were measured using a semiquantitative ELISA assay (Anti-SARS-CoV-2 ELISA IgA, Euroimmun). The results were evaluated by calculating the ratio between the extinction of samples and the extinction of the calibrator. The ratio was interpreted as follows: < 0.8 (negative), ≥ 0.8 to < 1.1 (borderline) and ≥ 1.1 (positive).

### 2.4 FASCIA assay

The FASCIA assay was performed as previously reported (12). In the assay, whole blood was diluted at 1:9 in RPMI supplemented with L-glutamine, penicillin and streptomycin. The blood/medium mixture was stimulated with a combination of overlapping peptides spanning the immunogenic domains of the SARS-CoV-2 spike, membrane and nucleocapsid proteins (0.6 nmol/mL; PepTivator SARS-CoV-2 prot S, PepTivator SARS-CoV-2 prot N, PepTivator SARS-CoV-2 prot M, Miltenyi Biotech, Germany) and *Staphylococcus aureus* enterotoxins A and B (SEA+SEB) (0.1 μg/mL each; Sigma-Aldrich, USA) or RPMI (unstimulated control) in sterile polypropylene Falcon 12 x 75 mm tubes to a final volume of 500 μL. As virus control, stimulation with peptide pools of a common cold coronavirus (HCoV-229E; 0.03 nmol/mL of each) (Peptides&Elephant, Germany) and CMV viral lysate (5 μg/mL; Microbix Biosystems Inc, Canada) were also used. The tubes were incubated for 7 days at 37°C, 5% CO_2_ and 95% humidity. After this time, the supernatant was collected and stored at −80 °C. The cells were subsequently stained with anti-CD3 PerCP-Cy 5.5, anti-CD8-V450, anti-CD4 APC-Cy7, anti-CD154 APC, anti-CD137 BV650 and anti-CD19 PE (BD Biosciences, USA). All the tubes were incubated for 10 minutes in the dark at room temperature. The erythrocytes were then lysed with IOTest 3 Lysing Solution 10X (Beckman Coulter, USA) and the samples were centrifuged at 1800 rpm for 5 minutes. The supernatant was discarded and the cells were resuspended in 450 μL of phosphate buffered saline with 1% of bovine serum albumin. Blast counts were acquired for 120 seconds in an LSRFortessa flow cytometer (BD Biosciences, CA, USA).

The absolute number of proliferating cells was calculated using a Trucount tube (BD Biosciences, CA, USA), as previously reported (12). The values of the unstimulated samples (with RPMI) were subtracted from the values of the stimulated samples.

### 2.5 Study of T-cell phenotype

PBMCs were isolated and cryopreserved. At the time of analysis, 5 x 10^5^ thawed PBMCs were incubated with the following monoclonal antibodies: anti-CD3 VioBright R720, anti-CD8 APC, anti-CD4 APC-Vio770, anti-CD45RA PE-Vio615, anti-CD57 Vioblue, anti-PD1 ViobrightFITC, anti-CD49d PE770 (Miltenyi Biotech, Germany), anti-CCR7 BV785 (Biolegend, USA) and FVS510 (Fixable Viability Stain 510) (BD Biosciences, USA).

After 10 min on ice in the dark and one wash with PBSA (PBS supplemented with bovine serum albumin), the cells were acquired in an LSRFortessa flow cytometer (BD Biosciences, CA, USA). Dot plots were generated using FlowJo v10.6.2 software (Tree Star, USA).

### 2.6 QuantiFERON monitor assay

The QuantiFERON Monitor assay (Qiagen, Germany) is an *in vitro* diagnostic test that detects cell-mediated immune function through the measurement of interferon gamma in plasma by enzyme-linked immunosorbent assay (ELISA) following incubation of whole blood with innate and adaptive immune response stimulants.

The QuantiFERON Monitor assay was performed with one milliliter of heparinized whole blood collected in specific tubes. Samples were stimulated with anti-CD3 (as T-cell stimulant) and R848 (as toll-like receptor 7 agonist) on single lyophilized spheres within 8 hours from blood sample collection. Stimulated blood samples were incubated for 16 to 24 hours at 37°C and then centrifuged to harvest the supernatant. To preserve the quality of the samples, the supernatants were stored at -80°C until ELISA was performed.

### 2.7 Statistical analysis

The statistical analysis was performed using IBM SPSS Statistics 24.0 software (SPSS Inc., Chicago, IL, USA). Categorical variables were compared using the Chi-square or Fisher tests. The quantitative variables were compared using the Mann–Whitney *U* (two groups) or Kruskal–Wallis (more than two groups) tests. ROC (receiver operating characteristic) curve analysis was used to assess the ability of the T-cell proliferation assay to discriminate between individuals with past COVID-19 infection and healthy controls. Values were considered statistically significant when the *p* value was < 0.05. Graphic presentation was performed with GraphPad Prism 7 (GraphPad Software Inc.).

## 3 Results

### 3.1 Patients

A total of 86 convalescent patients with a history of COVID-19 (RT-PCRpos) from the Reina Sofía Hospital (Cordoba, Spain) were enrolled in the study. Of the 86 patients, 59 (68.6%) had demonstrable IgG antibodies to SARS-CoV-2 whereas 27 (31.4%) patients had undetectable IgG at initial routine IgG testing (seronegative patients). Of the seropositive patients, 33 were asymptomatic or had mild disease (fever, cough, fatigue, runny nose or myalgia) and 26 had severe disease (hypoxemia, respiratory distress, requiring oxygen support). All the seronegative patients had mild disease. Thirty-three healthy control subjects who were not infected with SARS-CoV-2 nor vaccinated and had undetectable IgG were recruited. The median age of the patients was 47 years (range 23–70). The median time from the first positive PCR to the first routine IgG testing was 22 days (IQR 13–42 days). No significant differences were found among the three groups of patients (23 days for mild infection, 26 days for severe infection and 18 days for seronegative; *p* = 0.088). The time from the last positive PCR and the sample collection was 178 days (IQR 143–214 days). Most patients had cough, fever, headache or diarrhea at illness onset. The most common comorbidities were hypertension (24.4%), dyslipidemia (14%), obesity (14%), cardiovascular disease (11.6%) and respiratory disease (11.6%) (Clinical and demographic characteristics are shown in **Table 1**).

**Table 1:** Demographic and clinical characteristics of the 119 participants.

Characteristics	Mild (PCR+/IgG+) n=33	Severe (PCR+/IgG+) n=26	Seronegative (PCR+/IgG-) n=27		Control (PCR-/IgG-) n=33	
**Age (median, range)**	43 (23-66)	59 (33-70)	47 (28-70)		43 (24-67)	
**Sex, n (%)**						
**Male**	16 (48.5)	17 (65.4)	14 (51.9)		17 (51.5)	
**Female**	17 (51.5)	9 (34.6)	13 (48.1)		16 (48.5)	
**Days from 1^st^ PCR+ to 1^st^ IgG+ (median, IQR)**	23 (15-43)	26 (22.2-43.5)	18 (14-27)		–	
**Days from 1^st^ PCR+ to sample collection (median, IQR)**	175 (142-195)	182 (141-211)	203 (145-216)		–	
**Days from 1^st^ IgG+ to sample collection (median, IQR)**	132 (100-168)	139.5 (117.3-168)	166 (128-196)		–	
**CMV-seropositivity, n (%)**	24 (72.7)	25 (96.2)	22 (81.5)		23 (69.7)	
**Symptoms, n (%)**	28 (84.8)	26 (100)	16 (59.3)		0 (0)	
**Cough**	15 (45.5)	21 (80.8)	10 (37)		0 (0)	
**Fever**	15 (45.5)	23 (88.5)	8 (29.6)		0 (0)	
**Headache**	12 (36.4)	9 (34.6)	11 (40.7)		0 (0)	
**Diarrhoea**	7 (21.2)	18 (69.2)	5 (18.5)		0 (0)	
**Myalgia**	8 (24.2)	12 (46.2)	8 (29.6)		0 (0)	
**Dyspnea**	4 (12.1)	21 (80.8)	2 (7.4)		0 (0)	
**Odynophagia**	9 (27.3)	5 (19.2)	7 (25.9)		0 (0)	
**Ageusia**	13 (39.4)	4 (15.4)	3 (11.1)		0 (0)	
**Asthenia**	9 (27.3)	7 (26.9)	2 (7.4)		0 (0)	
**Anosmia**	8 (24.2)	5 (19.2)	4 (14.8)		0 (0)	
**Other**^**a**^	8 (24.2)	13 (50)	8 (29.6)		0 (0)	
**Oxygen supplement, n (%)**	0 (0)	26 (100)	0 (0)		0 (0)	
**Nasal glasses**	0 (0)	25 (96.2)	0 (0)		0 (0)	
**Reservoir mask**	0 (0)	4 (15.4)	0 (0)		0 (0)	
**Non-invasive ventilation**	0 (0)	4 (15.4)	0 (0)		0 (0)	
**Mechanical intubation**	0 (0)	1 (3.8)	0 (0)		0 (0)	
**Oxygen saturation, median % (IQR)**	–	92 (91-94)	–		–	
**Comorbidities, n (%)**						
**Hypertension**	5 (15.2)	12 (46.2)	4 (14.8)		3 (9.1)	
**Dyslipidaemia**	4 (12.1)	8 (30.8)	0 (0)		2 (6.1)	
**Obesity**	3 (9.1)	6 (23.1)	3 (11.1)		3 (9.1)	
**Cardiovascular disease**	4 (12.1)	5 (19.2)	1 (3.7)		3 (9.1)	
**Respiratory disease**	4 (12.1)	3 (11.5)	3 (11.1)		3 (9.1)	
**Diabetes**	3 (9.1)	4 (15.4)	1 (3.7)		0(0)	
**Other**	19 (57.6)	15 (57.7)	18 (66.7)		14 (42.4)	
**Cell-mediated immune function, (IU/mL IFNG; median, IQR)**^**b**^	348 (216-662)	372 (182-660)	351 (131-577)		451 (71-679)	

a Including runny nose, nasal congestion, nausea, discomfort, phlegm, seasickness, shivers, ganglion swelling or facial rash.

b Cell-mediated immune function was determined in a group of 87 participants.

Global non-specific cell-mediated immune function was evaluated in a subgroup of 87 participants and no statistical differences between the COVID-19 patients and controls (Mann–Whitney U test, *p* = 0.919) or among the three groups of patients were observed (Kruskal–Wallis test, *p* = 0.930) (**Table 1**).

### 3.2 Humoral response against SARS-CoV-2

**Figure 1:**
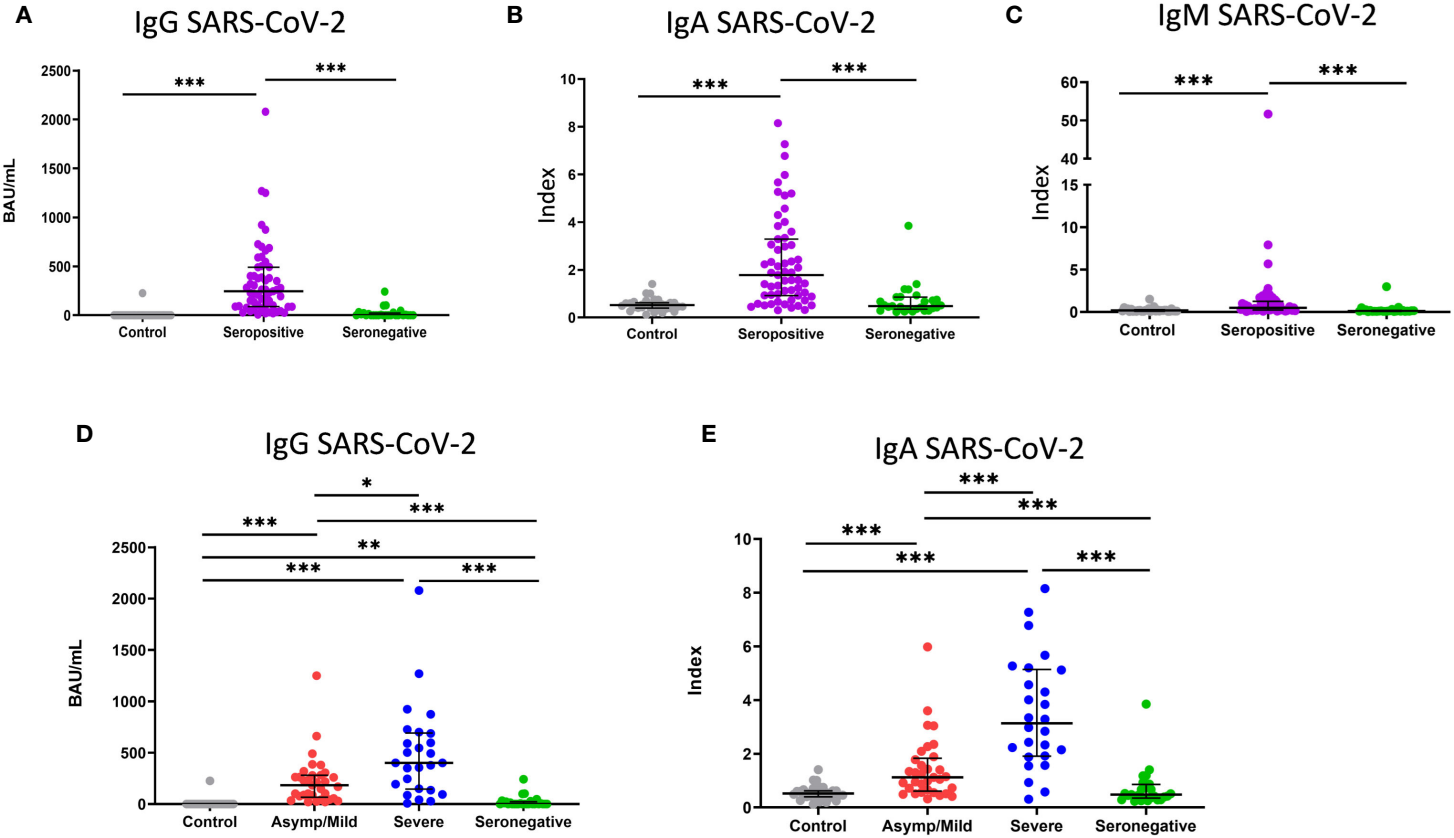
Level of IgG, IgA and IgM antibodies against SARS-CoV-2. **(A)** Binding antibody units mL (BAU/mL) of IgG, **(B, C)** index values of IgA and IgM in healthy controls (grey dots; n=33), seropositive patients (purple dots; n=59) and seronegative patients (green dots; n=27). **(D)** Binding antibody units per mL (BAU/mL) of IgG and **(E)** index value of IgA in healthy controls (grey dots; n=33) and asymptomatic/mild seropositive (red dots; n=33), severe seropositive (blue dots; n=26) and seronegative (green dots; n=27) patients. Each dot represents an individual. Median and IQR are shown. The Mann–Whitney U test was used. *<0.05, **<0.01, ***<0.001. Asymp/mild: asymptomatic or mild patients.

After comparing the re-tested SARS-CoV-2-specific IgG level with the initially obtained values, we found that more than 85% of initially seronegative participants were still seronegative at the time of sample collection (**Figure 1A**). IgM and IgA were then tested to evaluate whether seronegative patients also lacked SARS-CoV-2 IgA or IgM. As is shown in **Figures 1B, C**, seronegative patients had a lower median level of these two antibodies than seropositive patients but similar levels to the healthy controls.

We then analyzed whether there was any difference in the level of antibodies between mild or severe seropositive patients. The median levels of IgG and IgA were significantly higher in severe than in mild patients (401 vs. 184 BAU/mL for IgG, *p* < 0.005; 3.1 vs. 1.1 index for IgA, *p* < 0.001) (**Figures 1D, E**). No significant differences were found for anti-CMV IgG (data not shown).

### 3.3 T-cell response against SARS-CoV-2

**Figure 2:**
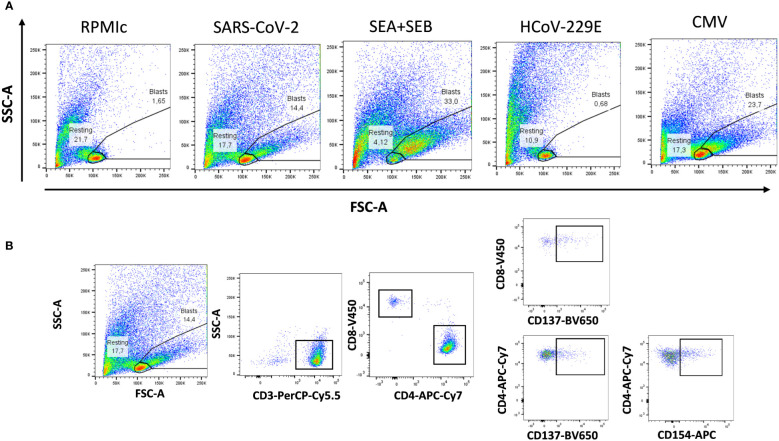
Flow cytometry gating strategies for analyzing proliferative reactivity of T cells. **(A)** Representative T-cell proliferation after whole blood stimulation with SARS-CoV-2, *Staphyloccocus aureus* toxins, common cold coronavirus HCoV-229E and CMV as well as unstimulated whole blood (medium) are shown. **(B)** Representative gating strategy for analyzing CD3+, CD4+ and CD8+ T-cell proliferation. Expression of activation markers (CD137 and CD154) was analyzed separately in CD4+ and CD8+T cells.

Subsequently, we evaluated the T-cell memory after stimulation with a combination of SARS-CoV-2 spike, nucleocapsid and membrane peptide pools using the FASCIA assay. In parallel, we also evaluated the proliferative capacity against superantigen SEA+SEB, HCoV-229E and CMV lysate. A representative plot of the proliferative response against antigens and medium and the gating strategy are shown in **Figure 2**.

**Figure 3:**
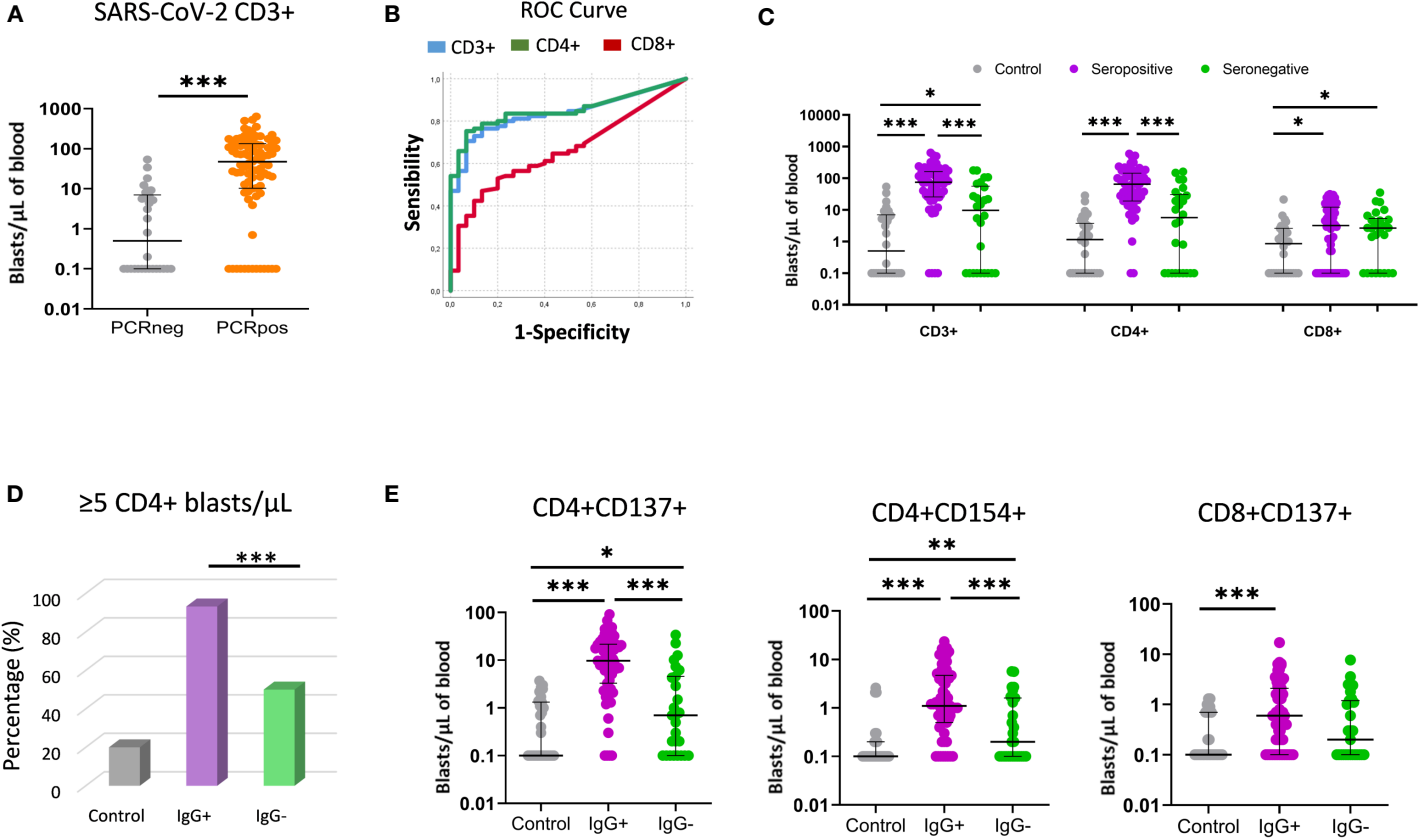
T-cell proliferative response using the FASCIA assay after stimulation with SARS-CoV-2 peptides. **(A)** Comparison of T-cell proliferative response between past infection patients (PCRpos) (orange dots; n=85) and healthy controls (PCRneg) (grey dots; n=30). Proliferating cells (blasts) are shown on a Forward Scatter (FSC) versus Side Scatter (SSC) dot plot. Median and IQR are shown. The Mann–Whitney test was used. **(B)** ROC curve analysis of CD3+ (blue line), CD4+ (green line) and CD8+ (red line) proliferative response to distinguish COVID-19 convalescent patients from healthy controls. **(C)** Comparison of CD3+, CD4+ and CD8+ response (blasts/µl) between healthy controls (grey dots; n=30), seropositive patients (purple dots; n=59) and seronegative patients (green dots; n=26). Median and IQR are shown. The Mann–Whitney U test was used. **(D)** Percentage (%) of participants with positive T-cell SARS-CoV-2 response (≥5 CD4+ blasts/µL) in healthy controls (grey bar), seropositive (purple bar) and seronegative (green bar) patients. **(E)** Comparative proliferation of CD4+CD137+, CD4+CD154+ and CD8+CD137+ T cells (blasts/µl of blood) between healthy controls (grey dots; n=30), seropositive patients (purple dots; n=59) and seronegative patients (green dots; n=26). Each dot represents an individual. Median and IQR are shown. The Mann–Whitney test was used. *<0.05, **<0.01, ***<0.001.

Three controls and one patient (4/119) had invalid proliferation because the assay did not function properly likely due to technical problems. The CD3+ blast response to SARS-CoV-2 was significantly higher in convalescent patients than in the controls (47.3 vs. 0.5 CD3+ blasts/µL of blood) (**Figure 3A**). To evaluate whether T-cell proliferation discriminated between individuals with past infection and healthy controls, a ROC curve analysis was performed for CD3+, CD4+ and CD8+ T cells. The area under the curve (AUC) indicated that CD3+ (AUC = 0.84) and CD4+ proliferations (AUC = 0.85) had a better discriminative capacity than CD8+ proliferations (AUC=0.66) (**Figure 3B**; **Table 2**). Cut-offs of 7 CD3+ blasts/µL of blood (80% sensitivity and 73.3% specificity) and 5 CD4+ blasts/µL of blood (80.2% sensitivity and 76.7% specificity) showed the best discriminatory capacity between convalescent PCR-confirmed patients and healthy individuals. Based on these data, we consider that patients with a T-cell proliferation ≥ 5 CD4+ blasts/µL had a “positive SARS-CoV-2 T-cell” response. According to this result, the 64.3% (74/115) of participants (20%, 6/30 of controls; 80%, 68/85 of patients) had a positive SARS-CoV-2 response at the time of sample collection. Very similar percentages were found when CD3+ instead of CD4+ response was considered.

**Table 2:** ROC curve for evaluating the capacity of T-cell reactivity (blast formation) to discriminate convalescent COVID-19 patients from healthy controls.

	AUC (95% CI)	*p*-value	Cut-off	Sensitivity	Specificity
CD3+	0.84 (0.77-0.91)	<0.001	7	0.80	0.73
CD4+	0.85 (0.78-0.92)	<0.001	5	0.80	0.77
CD8+	0.66 (0.56-0.76)	0.009	2.5	0.56	0.73

AUC, area under the curve.

#### 3.3.1 Seronegative versus seropositive patients

In this section we compare the T-cell proliferative response to SARS-CoV-2 peptides in seronegative versus seropositive patients. We observed that seronegative patients had a significantly lower T-cell reactivity than seropositive patients (9.6 vs. 74.5 CD3+ blasts/µL; *p* < 0.001) (**Figure 3C**). When CD4+ and CD8+ T-cell proliferation was analyzed separately, CD4+ T-cell proliferation was also observed to be lower in seronegative patients (5.6 vs. 64.9 CD4+ blasts/µL; *p* < 0.001), whereas no significant differences were detected for CD8+ T cells. Importantly, when the cut-off of ≥ 5 CD4+blasts/µL was considered, 93.2% (55/59) of seropositive patients had positive SARS-CoV-2 compared to only 50% (13/26) of seronegative patients (chi-square; *p* < 0.001) (**Figure 3D**).

In addition, the frequency of CD4+ blasts expressing the CD137 or CD154 activation markers were also significantly lower in seronegative patients (**Figure 3E**). No significant differences were observed between seronegative and seropositive peptides in the T-cell response to CMV lysate or HCoV-229E peptides (**Supplementary Figure 1**).

#### 3.3.2 Severe versus asymptomatic/mild seropositive patients

**Figure 4:**
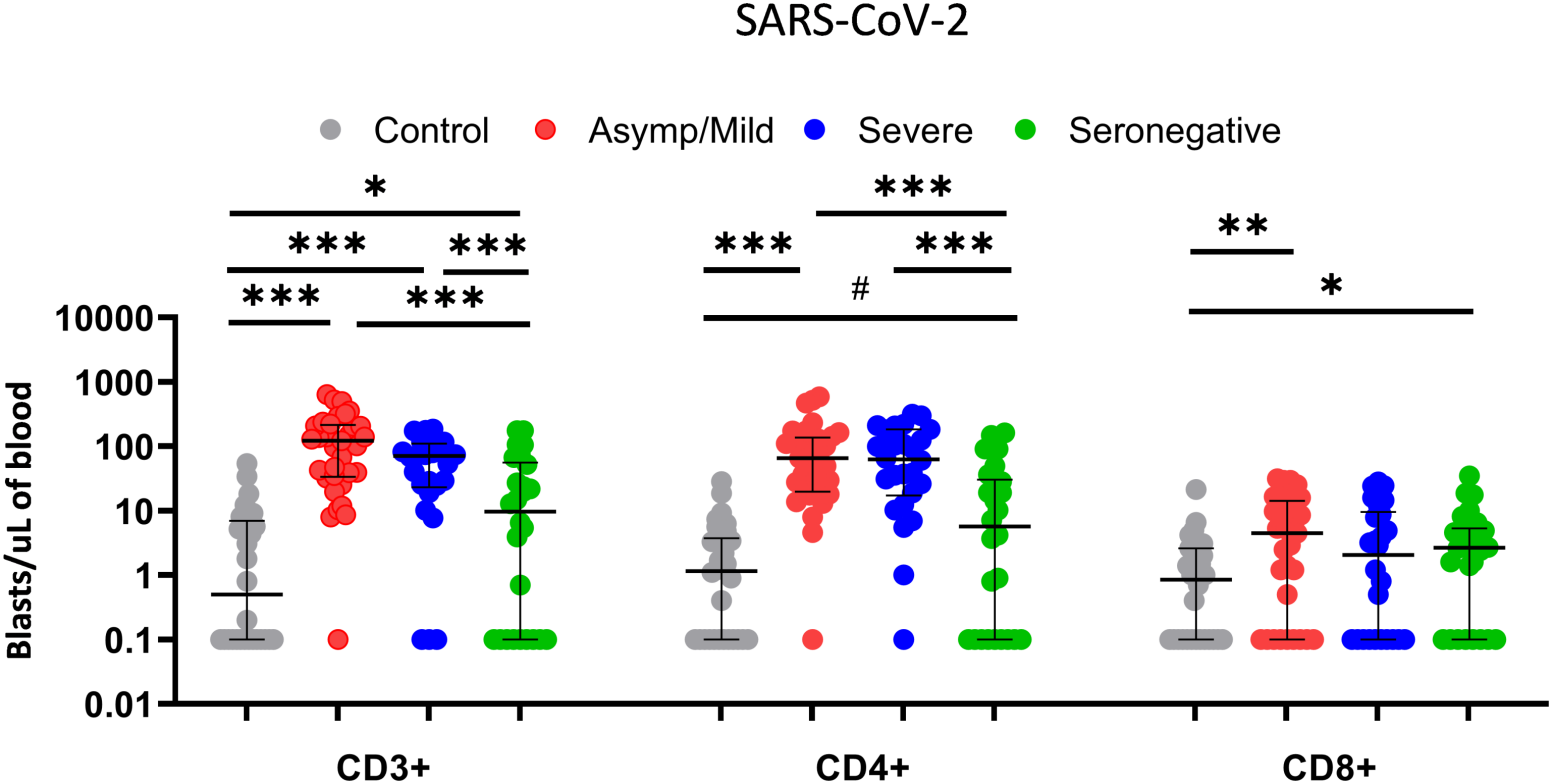
Comparison of T-cell proliferative response of CD3+, CD4+ and CD8+ T cells (blasts/µl of blood) between healthy controls (grey dots; n=30), asymptomatic/mild seropositive patients (red dots; n=33), severe seropositive patients (blue dots; n=26) and seronegative patients (green dots; n=26) after stimulation with SARS-CoV-2 using the FASCIA assay. Each dot represents an individual. Median and IQR are shown. The Mann–Whitney U test was used. # 0.054, *<0.05, **<0.01, ***<0.001.

We then analyzed T-cell reactivity against SARS-CoV-2 peptides in the subgroups of seropositive patients according to the severity of symptoms. No significant differences in T-cell proliferation were found between severe and mild patients, neither as CD3+ nor as CD4+ and CD8+ separately (**Figure 4**). No significant differences with respect to reactivity against HCoV-229E and CMV lysate were observed either (**Supplementary Figure 1**).

### 3.4 Comparison of memory T-cell phenotype

Next, we explored differences in the immune phenotype of memory T lymphocytes in the peripheral blood of seropositive and seronegative patients. We analyzed the expression of CD45RA and CCR7 on the surface of CD4+ and CD8+ T cells to identify the memory cells, as well as PD1 and CD57 as late-memory markers.

**Figure 5:**
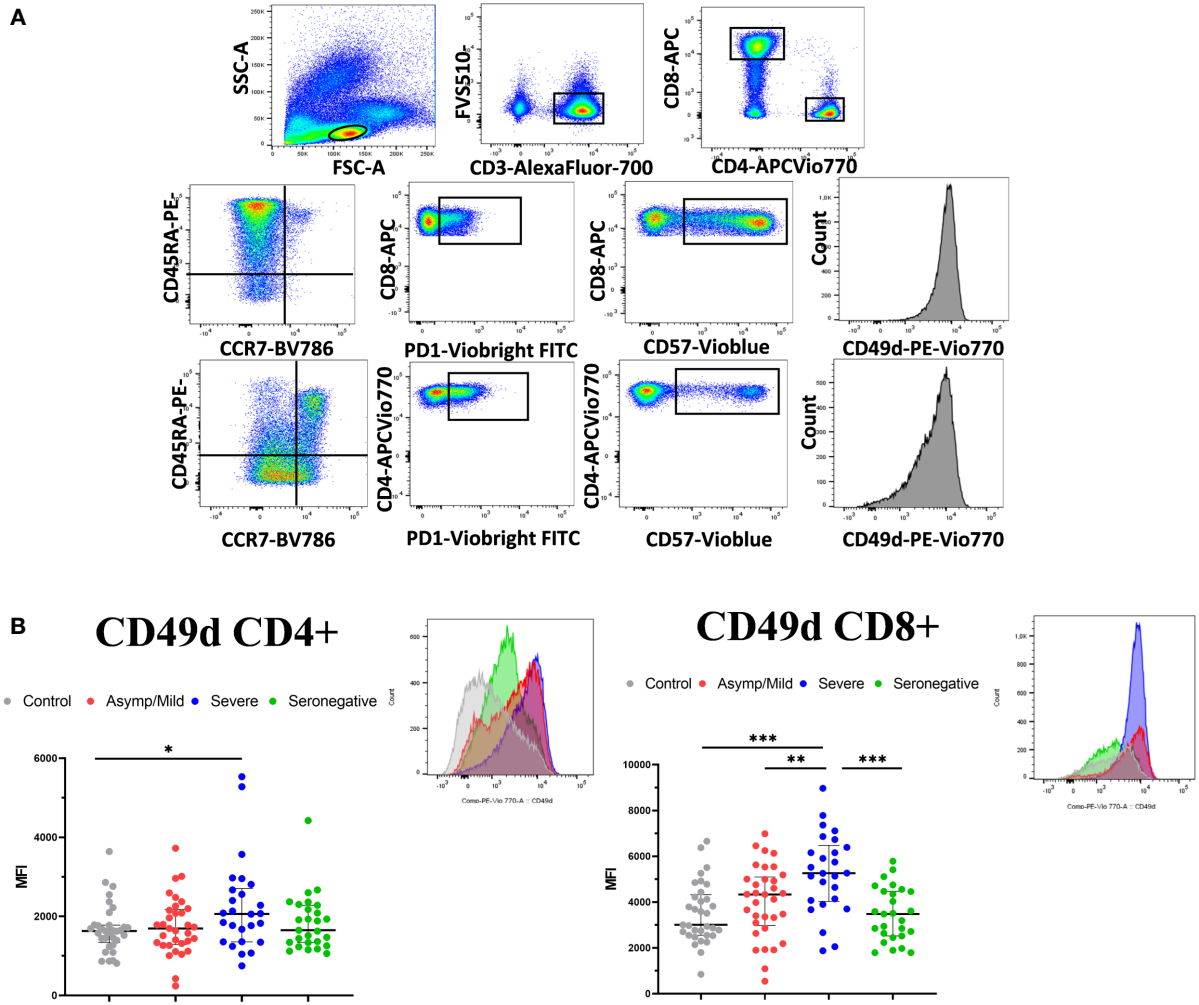
T-cell phenotypic characterization of PBMCs in convalescent COVID-19 patients and healthy controls. **(A)** Gating strategy for analyzing PBMC phenotype. Live CD3+ cells were selected and CD4+ and CD8+T cells were gated separately to analyze memory and naïve T cells (CD45RA/CCR7), expression of late memory markers (PD-1 and CD57) and integrin CD49d. **(B)** PBMCs were assessed for expression of CD49d on CD4+ and CD8+ T cells. Data represent the MFI. A representative histogram of each group is shown above the scatter plot. Median and IQR are shown. The Mann–Whitney test was used. *<0.05, **<0.01, ***<0.001.

We also assessed the expression of CD49d, an integrin involved in T-cell activation (**Figure 5A**). Interestingly, most differences were found for CD49d, which showed a significantly lower expression on CD8+ T cells in seropositive patients compared to the seronegative patients (median fluorescence intensity, 4643 vs. 3483; *p* = 0.003) (**Figure 5B**). In turn, CD49d expression on CD8+ T cells was higher in severe than in mild patients (median fluorescence intensity, 5262 vs. 4334; *p* = 0.008). No significant differences were observed between seronegative and seropositive patients with mild infection (median fluorescence intensity, 4334 vs. 3483; *p* = 0.119)

No other significant differences were found, although the percentage of CD4+ EMRA (CD45RA+CCR7-) cells tended to be higher in seronegative than in seropositive patients.

### 3.5 Vaccination and post-vaccination SARS-CoV-2 infection

Since most of the participants were vaccinated against COVID-19 some months after enrolling in the study, we then investigated whether having a positive SARS-CoV-2 response pre-vaccination provided higher protection against SARS-CoV-2 infection after vaccination. Fully vaccinated was defined as having received at least two doses of the Pfizer, Moderna or Astrazeneca vaccine or one dose of the Janssen vaccine. Partially vaccinated was defined as having received one dose of the Pfizer, Moderna or Astrazeneca vaccine. Of the 119 participants, 88 (73.9%) received the complete vaccination. Among the fully vaccinated participants, a higher incidence of post-vaccination infection was found in controls compared to patients (42.3% vs 19.4%; *p* = 0.025). However, no significant differences were observed in the reinfection incidence in COVID-19 patients according to serology status (18.6% of seropositive and 21.1% of seronegative patients; *p* = 1.000) or according to the T-cell response (30.8% in patients <5 CD4+ blasts/µL and 16.3% in patients with ≥5 CD4+ blasts/µL; *p* = 0.256) (**Table 3**).

**Table 3:** Incidence of post-vaccination SARS-CoV-2 infection in fully vaccinated participants.

	Fully vaccinated (n=88)	*p*-value
All (n=88)		
Healthy controls	11/26 (42.3)	0.025
COVID-19 patients	12/62 (19.4)	
COVID-19 patients (n=62)		
SARS-CoV-2 IgG response		
IgG-	4/19 (21.1)	1.000
IgG+	8/43 (18.6)	
SARS-CoV-2 T-cell response		
<5 CD4+ blasts/µL	4/13 (30.8)	
≥5 CD4+ blasts/µL	8/49 (16.3)	0.256

## 4 Discussion

This observational study analyzed SARS-CoV-2 T-cell response in a group of non-vaccinated convalescent COVID-19 patients, including not only mild and severe PCRpos IgGpos patients but also a group of patients with PCRpos but undetectable IgG. To assess T-cell response, we used the FASCIA assay, which measures T-cell proliferation after stimulation with a mixture of SARS-CoV-2 peptides. The main advantages of this method is that is based on whole blood and therefore does not require PBMC preparation, uses minimal equipment and can be suitable for clinical use (12). This study shows that 1) This proliferative assay is useful to discriminate between past infection patients and control individuals, with 5 CD4+ blasts/µL of blood being the best cut-off to discriminate both groups; 2) It is also useful for distinguishing seronegative from seropositive PCRpos patients, since the percentage of patients with positive SARS-CoV-2 T-cell response was significantly lower in the seronegative group, thus suggesting that impaired proliferation of this T-cell subpopulation and the lack of specific IgG might be related.

In this study, T-cell response was detected in 80% of convalescent patients more than 3 months after the last positive SARS-CoV-2 RT-PCR. This result is in line with data published by other authors, who have observed that the majority of recovered patients maintained SARS-CoV-2 T-cell responses for 6-8 months post-infection, suggesting that this cellular immunity is a long-lasting immunity and longer than humoral immunity (13–17). In addition, we detected T-cell reactivity in 20% of healthy controls, which is consistent with the results of Braun et al., who found pre-existent spike-reactive CD4+ T cells in 35% of healthy donors (18). Cross-reactivity has been described in multiple publications and a variable percentage of unexposed individuals with SARS-CoV-2 specific T-cell response has been reported (14, 19–22). Cross-reactivity could be related to previous immunization against common cold coronavirus infections. Although we did not find a relationship between *in vitro* T-cell reactivity against SARS-CoV-2 peptides in healthy controls and T-cell response against HCoV-229E, we cannot rule out the possibility that proliferation against other common coronaviruses such as HCoV-OC43, HCoV-NL63, HCoV-HKU1 may exist in controls (18, 23). Nevertheless, despite cross-reactivity, proliferative response had a good ability to discriminate between individuals with resolved infection and unexposed individuals. The number of CD4+ blasts discriminated patients from controls better than CD8+ blasts, since CD8+ proliferation was very low in both groups. This low CD8+ response is consistent with Eneksoon et al. (22), who reported low CD8+ reactivity with both SARS-CoV-2 peptide pool and whole viral particles using the same proliferative method.

Our study focuses on convalescent seronegative COVID-19 patients (PCRpos IgGneg), which represent a subgroup of PCR-confirmed infected patients with undetectable anti-SARS-CoV-2 IgG. The lack of anti-SARS-CoV-2 IgG in this subgroup might be related to the short time between infection and the initial IgG testing when antibodies were not produced in sufficient amounts (10). However, most of the seronegative patients continued lacking SARS-CoV-2 IgG several months later when they were recruited and the IgG was retested. Another explanation might be related to an insufficient SARS-CoV-2 viral load below the threshold to trigger humoral response. However, viral load data were not available at the time of recruitment and we could not evaluate this possibility. Nevertheless, the significantly higher proliferative T-cell response in seronegative patients compared to the controls indicates that, despite lacking humoral response, seronegative patients had SARS-CoV-2-specific memory T cells with proliferative capacity upon stimulation, although of lower magnitude than seropositive patients. This contrasts with the results published by Steiner et al., since they described a robust and comparable T-cell response in seronegative and seropositive patients (21).

The lower CD4+ T-cell response we found in seronegative patients does not appear to be related to a lower lymphocyte count in this group since no significant differences in the global immune function measured by the QuantiFERON-Monitor assay was observed among the three groups of patients. The impaired CD4+ T-cell response in seronegative patients might be related to their lack of IgG, as reported by Odendhal et al., who found a strong correlation between neutralizing IgG and Th1 CD4+ T cells in convalescent patients (15). In addition, the decreased CD4+ T-cell proliferation might also be related to the lower expression of the integrin CD49d on CD8+ and CD4+ T we found in seronegative patients, since a low expression of CD49d on T cells has been associated to impaired T-cell reactivity (24, 25). Consistently, the higher expression of CD49d on CD8+ T cells in severe patients might be related to a dysfunctional hyperactivation of these cells that could in turn be related to the severity of symptoms.

The presence of SARS-CoV-2 T-cell response in patients lacking humoral response due to immunodeficiency or immunotherapy has also been also reported (4, 26, 27). Gadani et al. reported robust SARS-CoV-2 T-cell response in multiple sclerosis patients on anti-CD20 therapy (28). Interestingly, SARS-CoV-2 T-cell response has even been detected in patients with primary antibody deficiency (agammaglobulinemia or common variable immunodeficiency), indicating that the lack of SARS-CoV-2 humoral immunity may be compensated by innate and T-cell immunity to prevent severe COVID-19 (29, 30). Nevertheless, discordance between humoral and cellular response is not limited solely to SARS-CoV-2 but has also been observed in other viral infections such as cytomegalovirus (31–33).

This study has important limitations. Firstly, the sample size is small, which might preclude obtaining significant associations. Secondly, it examines T-cell reactivity against spike, nucleocapsid and membrane peptides but approaches including other SARS-CoV-2 protein regions or whole viral particles could add further information. Another limitation is related to the virological data, since viral load data would help to understand if the lack of SARS-CoV-2 IgG antibodies in seronegative patients might be related to a low viral load that is insufficient to trigger humoral response.

In conclusion, the FASCIA proliferation assay is useful not only to discriminate past infection patients from healthy controls, but also to distinguish seropositive patients from PCR-confirmed patients with undetectable SARS-CoV-2 IgG antibodies. Memory T cells in seronegative patients are able to respond to SARS-CoV-2 peptides upon stimulation, although at a lower magnitude than in seropositive patients. However, it should be highlighted that, despite the lack of humoral response, the low reactivity of T cells in seronegative patients seems to provide similar protection against SARS-CoV-2 reinfection after vaccination to that observed in seropositive patients.

## Data availability statement

The raw data supporting the conclusions of this article will be made available by the authors, without undue reservation.

## Ethics statement

The studies involving human participants were reviewed and approved by Ethics Committe of the Reina Sofia University Hospital. The patients/participants provided their written informed consent to participate in this study.

## Author contributions

JT-C and SC contributed to the conception and design of the study. RF-M and ABP selected the study participants. RF-M and JV-A performed the experiments. AP-V, AS, and AC participated in the data collection. RF-M organized the database. SC performed the statistical analysis. All authors contributed to the manuscript revision, read and approved the submitted version.

## Supplementary material

Supplementary Figure 1
**(A)** Comparison of CD3+, CD4+ and CD8+ blasts/µl of blood between healthy controls (control) (grey dots; n=30), seropositive patients (purple dots; n=59) and seronegative patients (green dots; n=26) after stimulation with common cold coronavirus HCoV-229E and **(B)** CMV using the FASCIA assay. Each dot represents an individual. Median and IQR are shown. The Mann–Whitney U test was used. *<0.05, **<0.01, ***<0.001.
