# Supplementary figures and images for: Memory SARS-CoV-2 T-cell response in convalescent COVID-19 patients with undetectable specific IgG antibodies: a comparative study
Source: Front Immunol. 2023 Apr 26;14:1142918. doi: 10.3389/fimmu.2023.1142918 (PMC10169638; doi:10.3389/fimmu.2023.1142918)

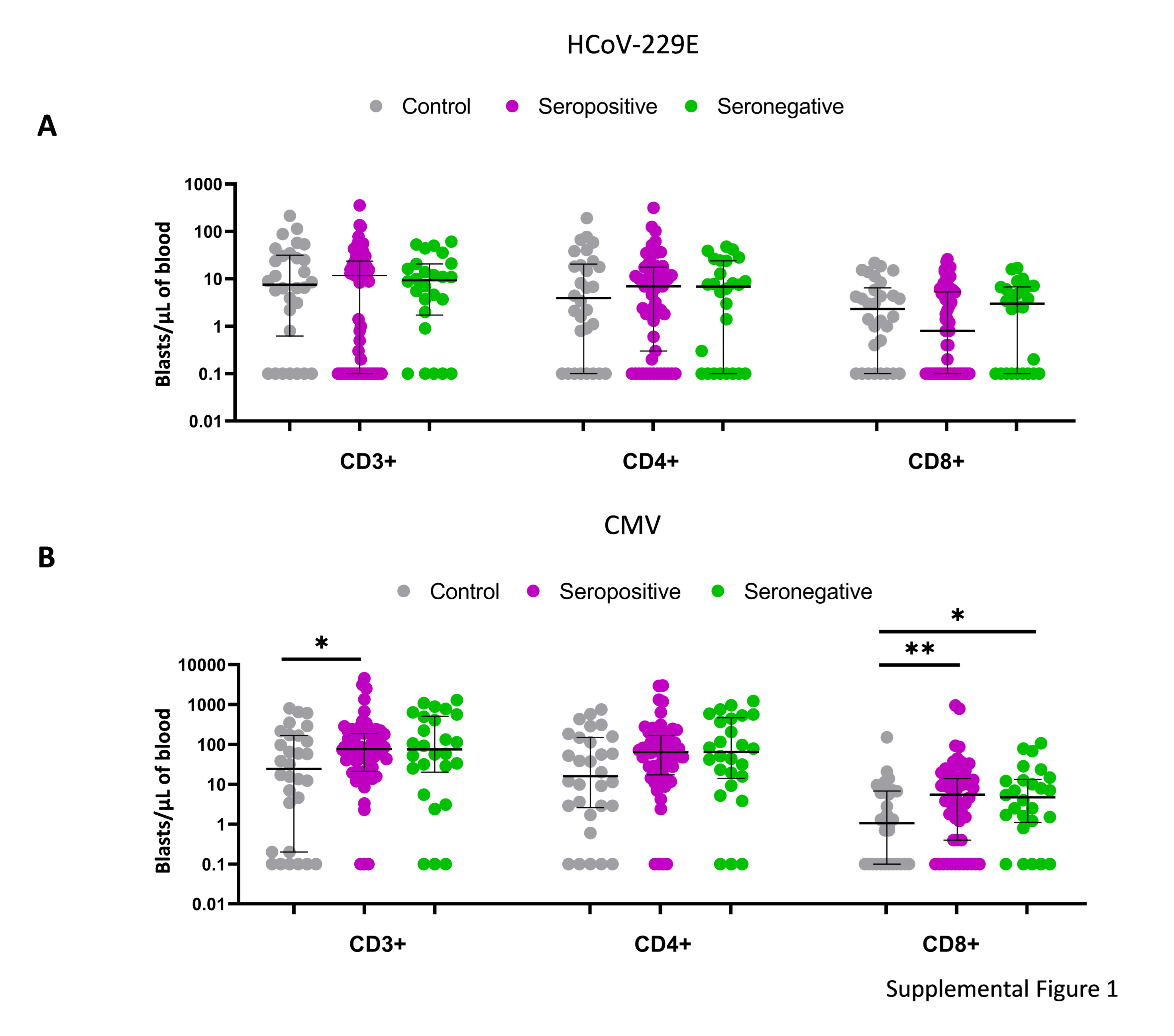

Supplement: Supplementary Figure 1 — (A) Comparison of CD3+, CD4+ and CD8+ blasts/µl of blood between healthy controls (control) (grey dots; n=30), seropositive patients (purple dots; n=59) and seronegative patients (green dots; n=26) after stimulation with common cold coronavirus HCoV-229E and (B) CMV using the FASCIA assay. Each dot represents an individual. Median and IQR are shown. The Mann–Whitney U test was used. *<0.05, **<0.01, ***<0.001. [file Image_1.tif]
